# Acoustic virtual walls enable open, scalable, and programmable microfluidics

**DOI:** 10.1126/sciadv.aec0104

**Published:** 2026-08-14

**Authors:** Hajin Oh, Mingyuan Liu, Tony Jun Huang, Junfei Li

**Affiliations:** ^1^ School of Mechanical Engineering, Purdue University, West Lafayette, IN 47907, USA.; ^2^ Thomas Lord Department of Mechanical Engineering and Materials, Duke University, Durham, NC 27708, USA.

## Abstract

Microfluidic platforms are widely used across biomedical research, chemical synthesis, diagnostics, environmental monitoring, and materials science for precisely manipulating small volumes of fluids and suspended particles. However, conventional systems rely on narrow physical channels that are prone to clogging, limited volumetric throughput due to high hydraulic resistance, and excessive shear stress that can damage sensitive cells and fragile materials. To overcome these constraints, we introduce acoustic channeling within a wide, open fluid chamber by replacing solid boundaries with acoustic virtual walls. These walls are formed by evanescent acoustic pressure fields generated from an engineered two-dimensional waveguide that suppresses internal wave propagation and produces highly localized subwavelength fields. This architecture minimizes shear stress while guiding particles along precisely defined trajectories. The electronically tunable acoustic field enables programmable, remote, and real-time particle control. Supported by simulations, we demonstrate diverse channeling designs, efficient particle collection, and material-specific separation. Operating at milliliter-per-minute flow rates, two orders of magnitude higher than conventional microfluidic systems, this platform enables scalable, clog-free microfluidics for high-throughput and robust applications.

## INTRODUCTION

Microfluidics has transformed chemical and biological analysis by miniaturizing fluidic processes down to the submicrometer and nanometer scales. Miniaturization has not only reduced reagent consumption but also enabled the development of assays with high resolution and sensitivity (*1*). These platforms have found broad application in biological studies, including droplet manipulation, drug delivery, and the creation of complex models such as organoids and cell culture systems (*2*–*11*). To further broaden functionality, microfluidics has spurred the development of advanced fluid and particle manipulation techniques that use external forces, such as electric, magnetic, or acoustic fields, to control target fluids/particles in the microenvironment (*12*–*22*). These noncontact approaches have enabled highly precise operations, including on-chip cell sorting, targeted drug encapsulation, and the assembly of multicellular constructs. As a result, microfluidics now underpins critical applications such as high-throughput single-cell profiling, organ-on-a-chip modeling, and controlled studies of cell-cell and cell-extracellular matrix interactions, providing unprecedented insight into physiological and pathological processes (*23*–*26*).

Despite the powerful capabilities for manipulating fluids and particles in controlled environments, the reliance on narrow microchannels in typical microfluidic devices introduces fundamental limitations. For instance, particles flowing through confined channels often adhere to channel walls or aggregate, causing persistent clogging that compromises device functionality, reduces reliability, and generates substantial platform waste (*27*–*29*). Throughput is similarly constrained: high hydraulic resistance in microchannels limits achievable flow rates and restricts their applications in large batches (*30*, *31*). The narrow channel also introduces high shear stress within the microchannel, which can damage cell components, such as cell membranes and cytoskeletal structures, jeopardizing the viability of the processed biological cells and other bioparticles (*32*–*35*). While techniques such as sheath flow and chemical surface treatments can help reduce the shear stress, they often suffer from increased system complexity and limited long-term biocompatibility due to surface degradation (*36*–*38*). These challenges underscore a critical need for microfluidic platforms that remove physical channel walls while preserving, or even expanding, the functional control traditionally provided by microchannels.

Acoustic tweezers have emerged as a novel technique for label-free, contactless, and precise particle manipulation with high biocompatibility (*12*). These capabilities have facilitated diverse applications, ranging from separating live and dead cells to enhancing cell encapsulation for drug delivery and high-purity bacterial filtration (*5*, *39*–*49*). In particular, resonant bulk acoustic wave (BAW) systems represent a well-established acoustofluidics paradigm that has previously demonstrated channeling, trapping, and sorting of the cells within fluidic environments (*50*–*52*). While these systems have established acoustic manipulation as a valuable tool in biological research, their reliance on fixed channel geometries and standing wave modes fundamentally limits the achievable field patterns and operational flexibility.

More broadly, existing acoustofluidic implementations based on holographic arrays, evanescent confinement, guided-wave devices, or streaming-assisted platforms have each made significant strides toward versatile particle manipulation. However, these approaches collectively face persistent challenges in simultaneously achieving deep-subwavelength resolution, dynamic field reconfigurability, and high-throughput operation within wide-open fluidic environments (*53*–*58*). Moreover, achieving higher spatial resolution generally necessitates operation at elevated frequencies, introducing substantial heat generation that can exceed the physiological tolerance of biological samples (*59*). Consequently, there remains a critical need for a platform capable of generating arbitrarily patterned, high-resolution acoustic landscapes at low frequencies within a wide-open fluidic environment.

In this study, we present a platform that replaces conventional physical microchannels with an acoustic virtual wall, using contactless ultrasonic manipulation to define dynamic pathways within a wide-open fluidic chamber. By engineering evanescent acoustic fields, our system generates high-resolution, customizable pressure landscapes at relatively low frequencies (200 kHz). Soft-material waveguides allow selective excitation of these fields with subwavelength precision, enabling precise control over fluidic environments. Beyond static guidance, the programmability of the acoustic virtual wall allows remote modulation of particle trajectories and temporal control over particle passage. Through a combination of numerical simulations and experimental validation, we demonstrate versatile functionalities, including precise channeling, programmable trapping, and material-specific sorting. These capabilities make the platform highly applicable for high-throughput assays and single-cell studies. More broadly, it offers a flexible approach for a wide range of microfluidic and lab-on-chip applications that require accurate, adaptable, and noncontact manipulation of particles or biological components.

## RESULTS

### Acoustic virtual walls for open-channel particle guidance

**Fig. 1.**
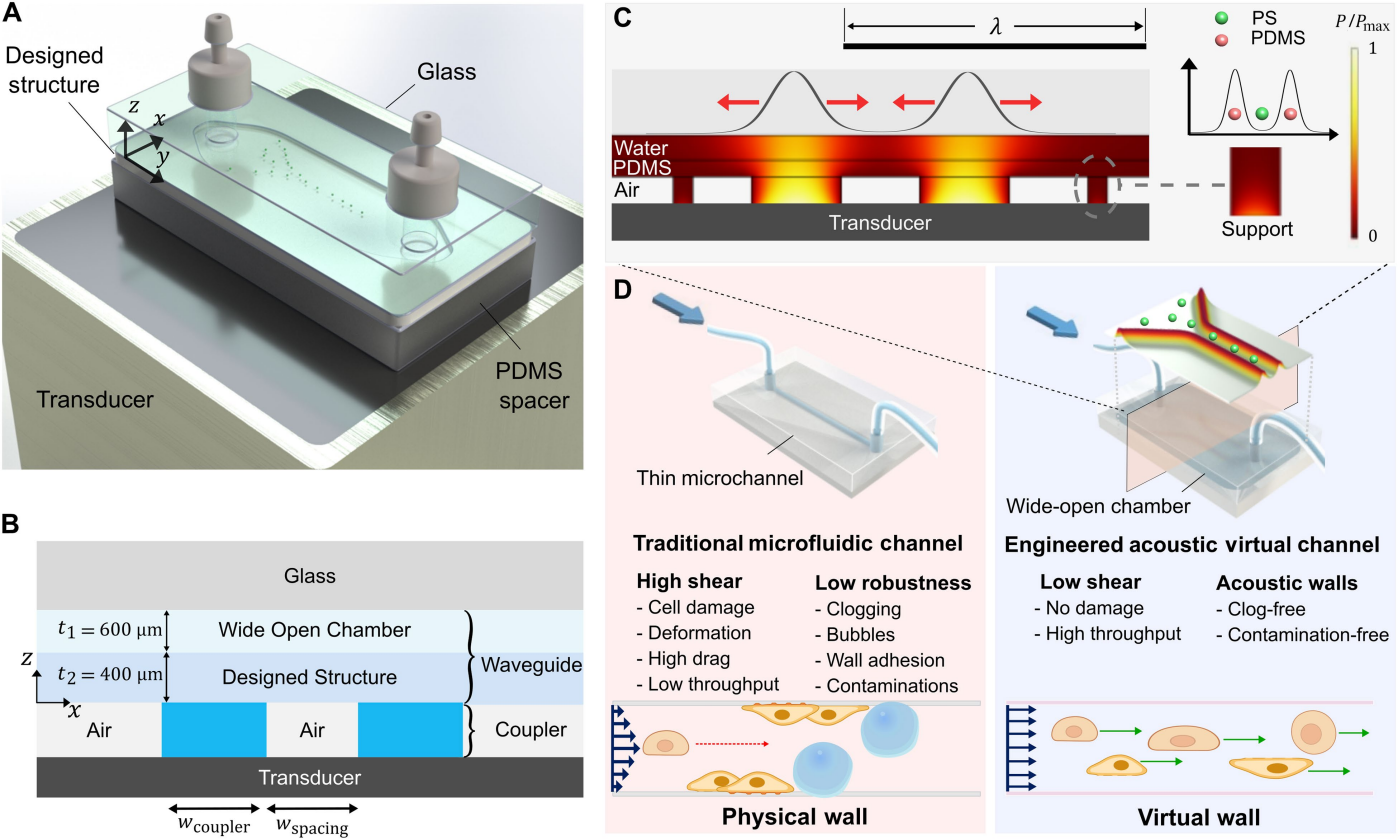
Illustration of acoustic virtual wall system. (**A**) 3D configuration of the acoustic virtual wall system. The particles are inserted from the inlet on the left and aligned following the acoustic virtual wall inside the wide water chamber. (**B**) Specific geometry of the acoustic virtual wall. The waveguide layer dimensions are selected to support an evanescent acoustic mode, and the couplers are patterned according to functional requirements. (**C**) The acoustic pressure field inside the system at 200 kHz. The acoustic pressure is evanescent in *x* direction that creates lateral pressure gradient for particle manipulation. The support width is chosen such that vertical wave number $k_{z}$ is imaginary and does not perturb the pressure field. (**D**) Conceptual comparisons between the traditional microfluidic channel and engineered acoustic virtual channel. Unlike conventional microfluidic channels confined by physical walls, the acoustic virtual channel supports wide-chamber with reduced shear and clog-free transport.

To address the fundamental limitations of narrow channels, we introduce acoustic virtual walls that guide particles along engineered trajectories within a wide-open chamber (Fig. 1A). By replacing physical boundaries with localized acoustic fields, we decouple particle guidance from physical boundaries. This platform leverages programmable acoustic pressure landscapes to enable arbitrary particle manipulation in an open-channel environment, effectively addressing the scaling and reliability bottlenecks of traditional microfluidics.

To achieve arbitrary acoustic walls in the water chamber, we designed a structure that localizes acoustic pressure along predefined patterns. The core of the system lies in the engineered waveguide layer and coupling layers (Fig. 1B). The waveguide layer dimensions are specifically designed to support an evanescent mode within, confining lateral sound propagation by leveraging the air boundary at the bottom surface. By patterning the couplers according to specific functional requirements, we can project a highly localized acoustic pressure field into the fluid medium without requiring physical walls. The evanescent pressure field, critically, remains highly localized with deep-subwavelength resolution beyond the diffraction limit. This property allows for arbitrary patterning with deep-subwavelength features.

The coupler layer consists of a coupling mask that bridges the ultrasonic source (transducer) to the waveguide layer. This mask consists of two types of units with distinct primary functions: a coupler that transmits the acoustic wave and a purely mechanical support. The key parameter that distinguishes a functional coupler from a purely mechanical support is the width of the unit, *w* (Fig. 1B). The vertical wave number, $k_{z}$ supported within the coupling layer, can be described by

$$k_{z}=\sqrt{{\left(\frac{2\pi f}{c}\right)}^{2}-{\left(\frac{\pi }{w}\right)}^{2}}$$ (1)

Below the critical width at a given frequency, a smaller *w* results in an imaginary $k_{z}$, characterizing a vertically evanescent pressure field (*60*). Detailed numerical simulations of the transmitted pressure field as a function of the coupler width, *w*, can be found in the Supplementary Materials.

Figure 1C shows the simulated pressure field of the overall system. The geometry is designed for 200 kHz, whose wavelength in water is approximately 7.5 mm. Assuming a uniform vertical incident wave and a negligible in-plane wave number, $k_{y}$, the acoustic pressure becomes highly localized within the coupler regions. The acoustic field originating from the mechanical support regions exhibits negligible perturbation radiating into the water channel, two orders of magnitude lower than those produced by the couplers. The mathematical representation of the pressure field inside the system is included in the Supplementary Material.

The resulting numerical simulations clearly demonstrate the capability for precise particle manipulation in the subwavelength regime. As illustrated in the schematic of particle interactions with the pressure field in Fig. 1C, particles move toward either the pressure maxima or minima based on their mechanical properties. As a demonstration, we compared the responses of two particle types with contrasting acoustic properties: polystyrene and polydimethylsiloxane (PDMS), both characterize a density similar to water. Polystyrene particles, which are acoustically stiffer than water, migrate toward pressure minima. In contrast, PDMS particles, which are acoustically softer than water, migrate toward the pressure maxima. The corresponding control resolution can be determined with either coupler’s width or spacing between the couplers, each of which in Fig. 1 (B and C) is $w_{\text{coupler}}\approx 0.3\;\lambda$ and $w_{\text{spacing}}\approx 0.25\;\lambda$, respectively. By exploiting these opposing acoustic responses, we designed and experimentally demonstrated multiple configurations of acoustic virtual walls capable of directing these different particle types along distinct trajectories within the wide water chamber. The detailed calculations of the acoustic radiation force are presented in Materials and Methods and the Supplementary Materials.

As illustrated in the conceptual comparison in Fig. 1D, this “virtual channel” represents a fundamental departure from conventional microfluidics. Unlike traditional channels, which are limited by the no-slip condition at physical walls, the acoustic virtual channel supports a wide-chamber architecture with significantly reduced shear stress. By moving particles away from high-friction boundaries and eliminating physical bottlenecks, the system ensures clog-free transport and maintains high-throughput operation even for high concentration analytes.

To validate the concept of a localized acoustic field within the waveguide layer, we developed a full 3D model of the platform supported by numerical simulations and experimental validation. PDMS was selected as the structural material due to its low shear modulus, minimal acoustic mode conversion, and excellent biocompatibility (*61*). We patterned the bottom surface of the PDMS layer with coupling masks tailored according to the target particle type and desired functionality. Above the PDMS, a wide-open water region served as the particle manipulation region, with dimensions larger than the wavelength λ. The top water chamber was subsequently sealed with a 6-mm-thick glass plate incorporating dedicated inlets and outlets. Last, to mitigate distorted near-field effects from the transducer, a spacer was incorporated between the transducer and the patterned PDMS structure. The spacer thickness was carefully designed to minimize spatial phase and amplitude variations associated with near-field propagation, thereby promoting the formation of a uniform and planar wavefront at the coupler interface. Details of this design are provided in the Supplementary Materials. With this platform established, we then evaluated four distinct coupling-mask configurations, each designed to demonstrate a specific functionality of the acoustic virtual walls. Detailed information about the fabrication process is included in Materials and Methods.

### Channeling for polystyrene particles

For particles stiffer than the surrounding water, such as polystyrene, the acoustic radiation force ($F_{\text{rad}}$) directs the particles toward the minimum of the Gor’kov potential ($U_{\text{rad}}$) (*62*, *63*)

$$F_{\text{rad}}=-\nabla U_{\text{rad}}$$ (2)

**Fig. 2.**
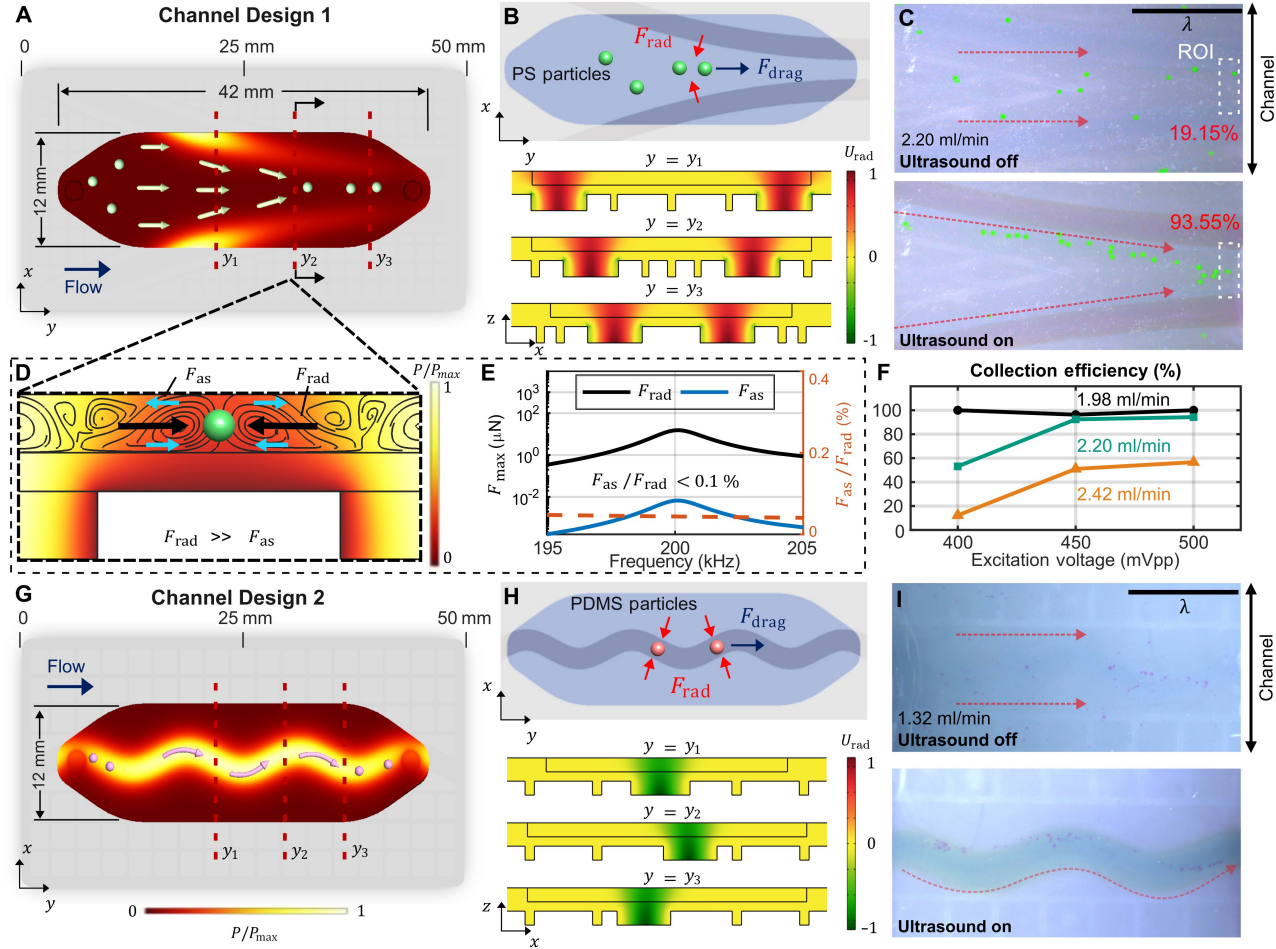
Acoustic virtual wall system for particle channeling. (**A**) Channel design for polystyrene (PS) particles. The acoustic pressure field engineered to guide PS particles into a straight channel using the funnel-shaped coupler patterning. (**B**) Forces and Gor’kov potential for PS particles. A lateral pressure gradient created inside the channel drives the PS particles to the channel center, while the drag from the flow carries particles in *y* direction. The acoustic radiation force is given by $F_{\text{rad}}=-\nabla U_{\text{rad}}$, directing particles toward the Gor’kov potential minimum. (**C**) Experimental results of channel design 1. At the flow rate of 2.20 ml/min, the acoustic virtual wall collects PS particles at the channel center, increasing the number of particles through the region of interest from 19.15 to 93.55%. (**D**) Schematic of forces in the acoustic virtual wall system. The black arrow denotes the acoustic radiation force ($F_{\text{rad}}$), while blue arrow represents the acoustic streaming-induced drag force ($F_{\text{as}}$). (**E**) Comparison of acoustic radiation and acoustic streaming force as a function of frequency. Streaming-induced force remains below 0.1% of acoustic radiation force over the operating frequency, with resonance behavior observed near 200 kHz due to the spacer geometry. (**F**) Collection efficiency as a function of excitation peak-to-peak voltage at different flow rates. As the flow rate increases, collection efficiency decreases, reaching approximately 60% at 2.42 ml/min with 500 mVpp excitation. (**G**) Channel design for PDMS particles. The acoustic pressure field is configured to route PDMS particles through a meandering channel. (**H**) Forces and Gor’kov potential for PDMS particles. Because of mechanical properties, PDMS particles experience radiation forces toward regions of higher-pressure amplitude. The Gor’kov potential gradient directs particles along the engineered pressure maxima. (**I**) Experimental results of channel design 2. At a flow rate of 1.32 ml/min, PDMS particles follow the meandering channel path created by the acoustic virtual wall.

As shown in Fig. 2 (A and B), we engineered a funnel-shaped coupler pattern that creates a lateral pressure gradient along the *x* axis and channels the particles to the center of the chamber.

This design consists of two adjacent couplers patterned as quarter ellipses, which create a localized pressure minimum. Figure 2B illustrates the forces acting on the particles, where the acoustic radiation force guides the particles, and the drag force directs them toward the outlet. With the given pressure gradient, the Gor’kov potential is mapped on three different cross sections that gradually channel the particles to the center. The minimum intercoupler spacing was 2 mm, which corresponds to a control resolution of approximately 0.27 λ.

Movie S1 and Fig. 2C show the experimental results, conducted using 250 to 300 μm fluorescent polystyrene particles flowing from the left inlet to the right outlet. At a flow rate of 2.20 ml/min, the particles were successfully aligned and channeled when the ultrasound was activated. Quantitatively, the number of particles passing through the defined region of interest increased sharply from 19.15% (ultrasound off) to 93.55% (ultrasound on).

To confirm that the observed channeling behavior is governed by the acoustic radiation force rather than acoustic streaming, we evaluated the relative contributions of these two effects using numerical simulations (*64*). As shown in Fig. 2D, the streaming field generated by the evanescent acoustic wave within the water channel exhibits a characteristic flow pattern along the pressure gradient. The resulting streaming-induced force on a particle, estimated from the Stokes drag associated with the streaming velocity field, acts in a distinct direction from the acoustic radiation force (Fig. 2D). The magnitudes of these two forces were then quantitatively compared across the evaluated frequency range (Fig. 2E).

Both forces exhibit a resonant enhancement at a characteristic frequency, attributed to the acoustic resonance of the PDMS spacer that amplifies the transmitted acoustic pressure into the water channel as discussed above. Notably, the ratio between the two forces remains approximately constant across the evaluated frequency range, demonstrating that the dominance of acoustic radiation force over streaming is robust and independent of the resonant behavior of the system. Across the evaluated frequency range, the streaming-induced force remains below 0.1% of the radiation force (Fig. 2E), confirming that particle dynamics in the present system are dominated by the acoustic radiation force. On the basis of this force balance, the critical radius below which acoustic streaming dominates over radiation force is estimated to be $R_{\text{critical}}\approx 2.62$ μm, well below the target particle size used in this study (*65*). Detailed calculation of critical radius is provided in the Supplementary Materials.

The present evanescent field framework can be extended to higher frequency regimes (1 to 4 MHz) through proportional geometric scaling. In fig. S5, the acoustic radiation and streaming-induced drag forces were calculated across frequencies by scaling the coupler dimensions (width and height) together with water channel height to preserve the acoustic field characteristics. The forces were calculated for a 10-μm particle, reflecting the reduced water channel height (down to 30 μm) in the scaled system. Despite the reduced dimensions, the acoustic radiation force remained dominant over the streaming-induced drag force across the investigated frequency range, indicating that effective particle confinement can be maintained under scaled operating conditions.

The system’s performance was systematically characterized at different flow rates as a function of input voltage to the transducer. As shown in Fig. 2F, at flow rate of 1.98 ml/min (corresponding to a mean cross-sectional velocity of 4.58 mm/s), the collection efficiency approaches nearly 100% across the tested voltage range, confirming robust particle confinement under moderate flow conditions. At a higher flow rate of 2.20 ml/min (5.09 mm/s), collection efficiencies of 92.41 and 94.26% were achieved at input voltage of 450 and 500 mVpp, respectively. At further elevated flow rate of 2.42 ml/min (5.60 mm/s), the efficiency dropped below 60%, reflecting the increased hydrodynamic drag that competes with the acoustic radiation force, placing this condition near the escape velocity limit of the present system. Given that the demonstrated flow rates already exceed those of conventional channel-confined microfluidic systems by more than an order of magnitude, operation within the characterized voltage range is sufficient for the intended high-throughput applications. It is noted that driving voltages beyond this range were found to compromise the structural integrity of the coupler-spacer interface, though such conditions are not required for the target operating regime. To further quantify this operational limit, escape velocities at different operating frequencies are evaluated in fig. S6, where the system maintains an escape velocity above 1 mm/s over a frequency range of 198.2 to 202.3 kHz, confirming robust virtual wall operation across the operating regime.

### Channeling for PDMS particles

Microfluidic channels often require complicated geometries; this design demonstrates our ability to create nonlinear virtual paths. Here, we used a single coupler to manipulate PDMS particles, which are mechanically softer than water, thereby attracting them to regions of high-pressure amplitude, as shown in Fig. 2G.

To visualize the effectiveness of the acoustic virtual wall in complex patterns, we configured a meandering channel based on a cosine wave profile. Figure 2H shows that the acoustic force created by the evanescent field collects the dispersed particles toward the center of the coupler and guides them along the meandering pathway. Along the center of this wavy path, local pressure maxima were created with a spatial resolution of approximately 0.24 λ. Despite the complexity of the geometry, experimental results at a flow rate of 1.32 ml/min show that the PDMS particles precisely follow the meandering virtual wall (Fig. 2I and movie S2). Unlike the dispersed trajectories seen under normal flow, the ultrasound-activated system efficiently collected the PDMS particles starting at the inlet and accurately guided them along the predefined wavy pattern.

### Programmable particle collection and release

A unique advantage of the acoustic virtual wall is its ability to serve as a dynamic collector under continuous flow. Unlike physical traps, this “virtual” trapping can be toggled electronically and adjusted to control particle aggregation.

**Fig. 3.**
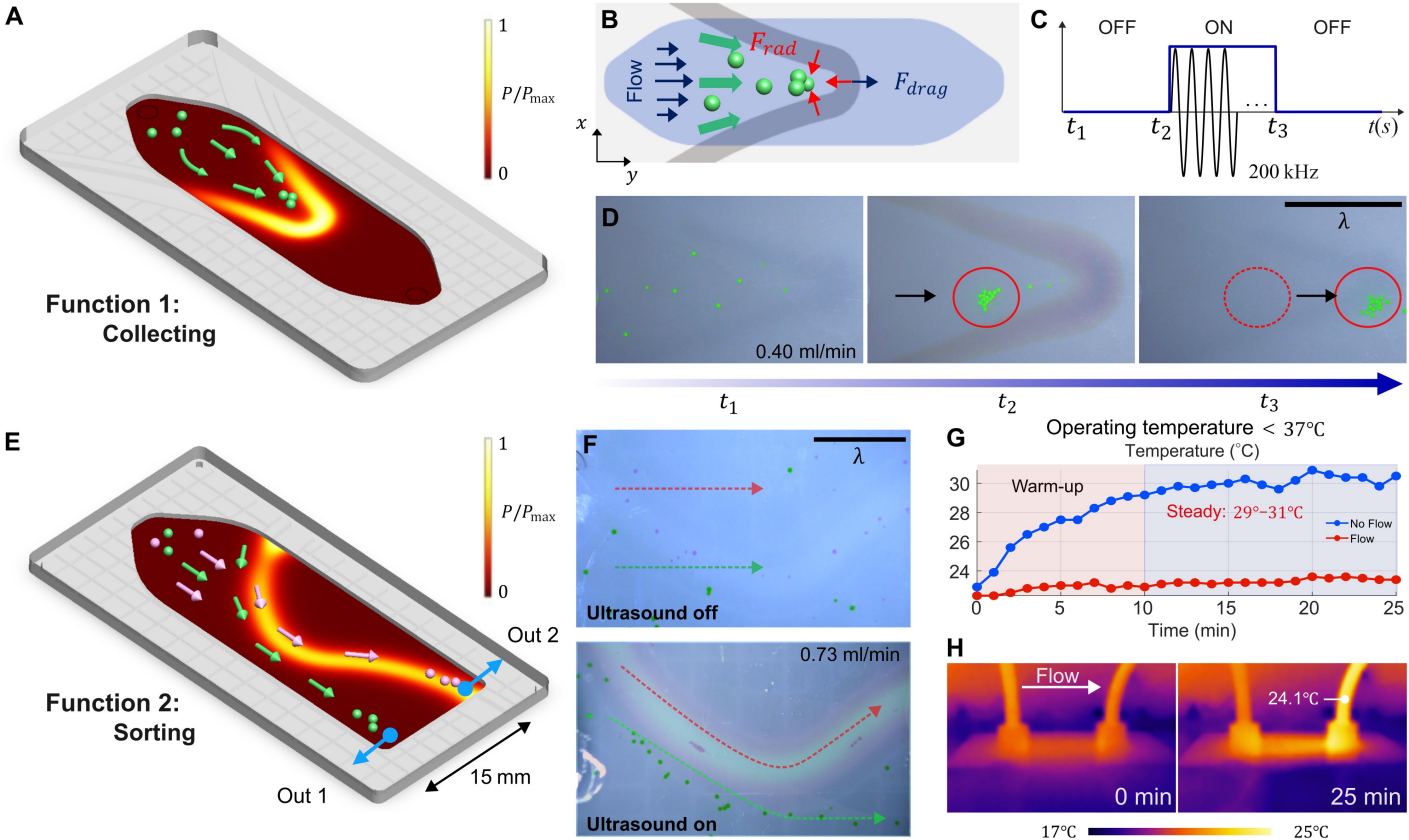
Advanced functionalities enabled by the acoustic virtual wall. (**A**) Collector design for PS particles. The acoustic virtual wall is configured to collect PS particles by balancing the radiation force against drag from the flow. (**B**) Collection mechanism. The radiation force traps incoming particles near the virtual wall, where they accumulate until release. (**C**) Programmable collection with automatic on-off control. Ultrasound activation at $t_{2}$ enables particle collection, and deactivation at $t_{3}$ releases the accumulated particles. (**D**) Experimental demonstration. PS particles converge to the channel center during ultrasound excitation at $t_{2}$, where they accumulate. Upon deactivation at $t_{3}$, the aggregated particles are released and advected downstream. (**E**) Sorter design for PS and PDMS particles. The acoustic virtual wall is engineered such that PS and PDMS particles experience different signs of acoustic radiation force, enabling separation at two distinct outlets. (**F**) Experimental results of sorter design. Under a flow rate of 0.73 ml/min, PS particles are expelled from the virtual wall and carried to outlet 1, while PDMS particles are guided along the virtual wall toward outlet 2. (**G**) Measured temperature of the platform during ultrasound excitation with and without the flow. The device temperature increases during ultrasound excitation and reaches steady state between 29° and 31°C without the flow and 24°C with the flow. (**H**) Surface temperature of the platform with the flow. The operating temperature remains below 37°C, within typical limits for biological applications.

The designed collector system traps polystyrene particles by generating an acoustic field that draws them into virtual cavities. Because of the fundamental difference in scaling, the acoustic radiation force is proportional to the particle radius cubed ($R^{3}$), while the hydrodynamic drag force is proportional to the radius (*R*). Therefore, the trapping efficiency is significantly magnified for large and stiff particles. The schematic of flow and the influence of acoustic virtual walls can be found in Fig. 3B, where the local collection region is 0.33 λ.

As shown in the temporal analysis (Fig. 3, C and D, and movie S3), the system remains passive until ultrasound activation at time $t_{2}$. Upon activation, the acoustic radiation force traps incoming polystyrene particles, causing them to accumulate at the virtual wall interface. From $t_{2}$ to $t_{3}$, polystyrene particles could be collected until they are released by deactivating the field at $t_{3}$. This programmable “on-off” control allows for the synchronized enrichment and release of analytes without mechanical valves.

### Binary sorting

The system’s ability to generate distinct force profiles for different materials enables high-purity sorting of polystyrene and PDMS particles (Fig. 3E). In this configuration, a single coupler stretches across the wide chamber, designed to simultaneously attract PDMS particles (acoustically soft) and expel polystyrene particles (acoustically stiff) as shown in Fig. 3 (E and F). We integrated two distinct outlets at the end to collect the separated particles.

Experimental results at a flow rate of 0.73 ml/min confirm clear spatial bifurcation of the two species, providing a method for the continuous-flow separation (movie S4). PDMS particles were effectively attracted to the coupled regions and channeled toward outlet 2, while polystyrene particles were expelled from the coupled region and exited through outlet 1.

A critical factor in integrating acoustic tweezers into the life sciences is heat dissipation management. We monitored the device temperature during continuous ultrasound excitation (Fig. 3, G and H). The temperature reaches a steady-state value between 29° and 31°C, remaining well below the 37°C threshold typical for maintaining the viability of sensitive biological samples. This thermal stability confirms that the acoustic virtual wall is a safe platform for handling live cells and temperature-sensitive reagents.

### Tunable reconfiguration of acoustic virtual wall systems

**Fig. 4.**
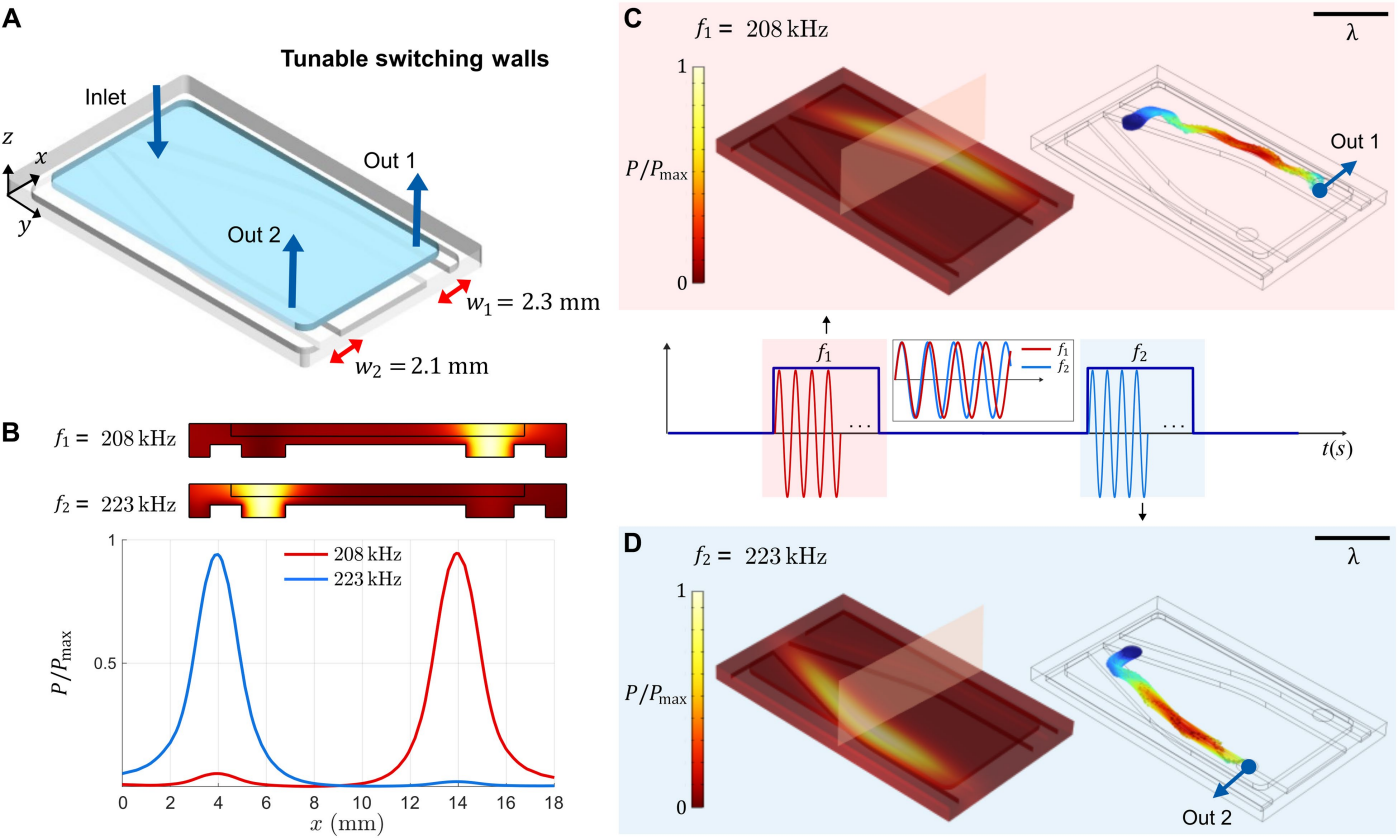
Tunable acoustic virtual wall via frequency control. (**A**) Schematic of tunable acoustic virtual wall. The coupler width is designed to target different resonant frequencies, enabling frequency-dependent routing of PDMS particles. (**B**) Cross-sectional pressure fields. Acoustic pressure fields at different excitation frequencies showing that wider coupler corresponds to lower resonant frequency and exhibit higher relative pressure amplitude under resonance. (**C**) 3D field and corresponding particle tracing at 208 kHz. At 208 kHz, the simulated pressure field directs PDMS particles along the upper virtual wall and toward outlet 1. (**D**) 3D field and corresponding particle tracing at 223 kHz. At 223 kHz, the pressure field redirects particles along the lower virtual wall and toward outlet 2.

Frequency-selective excitation enhances the adaptability of the acoustic virtual wall by incorporating geometry-dependent resonance into the system design. While preserving the evanescent-field-based guiding mechanism, we exploited resonance frequencies governed by the coupler width to selectively activate specific virtual wall paths within the same fluidic chamber, enabling real-time rerouting of particles (Fig. 4A). Each coupler resonates at a distinct frequency, such that a single acoustic source, operated without phase control and tuned only in frequency, defines the pathway for acoustically soft particles.

The operating principle relies on the relationship between coupler geometry and resonant frequency. As illustrated in Fig. 4B, cross-sectional analysis reveals that a wider coupler is tuned to a lower resonant frequency, where it exhibits significantly higher relative pressure amplitudes than nonresonant structures. This allows the user to “power on” specific sections of the virtual wall by matching the excitation frequency to the target coupler’s geometry.

We demonstrated this tunability by routing PDMS particles between two distinct outlets using frequency shifts. At 208 kHz, the pressure field is concentrated along the upper virtual wall. Particle tracing simulations of the given pressure field confirm that PDMS particles, attracted to pressure maxima, follow an upper trajectory and exit through outlet 1 (Fig. 4C). By shifting the excitation to 223 kHz, the acoustic pressure field redirects the particles toward the lower virtual wall. The resulting pressure field successfully reroutes the particles toward outlet 2 (Fig. 4D).

This frequency-control mechanism eliminates the need for complex integrated microvalves or mechanical switches, minimizing the risk of mechanical failure and significantly simplifying the fabrication and operation of complex sorting systems. A single wide channel can serve as a multipathway routing platform, where the destination of the analytes is determined solely by the excitation frequency of the acoustic transducer. Beyond discrete frequency switching, further incorporation of the acoustic metamaterial concepts through structural modification of the coupler geometry could enable high-quality-factor resonance, facilitating efficient energy localization, reducing the required acoustic power, and broadening the scope of biocompatible operation.

Furthermore, the integration of active array system would allow direct, real-time control of the acoustic field distribution. By aligning the coupler pattern with individual transducer segments with specific coupler branches, the system could dynamically reconfigure acoustic virtual walls without relying on frequency switching or structural modification, enabling truly arbitrary and on-demand spatial field patterning.

## DISCUSSION

In this work, we have demonstrated an acoustic tweezer platform capable of generating pressure field patterns that guide, trap and separate particles at subwavelength resolution. This system achieves its functionality through the innovative use of an evanescent acoustic field, which enables the formation of localized pressure within a wide-open water chamber, effectively creating acoustic virtual walls and eliminating the need for physical microchannel boundaries. Supported by optimized coupler geometries, we showcased four distinct designs of acoustic virtual walls. These configurations robustly demonstrated particle control at a resolution of one-quarter of the acoustic wavelength and operated reliably at high flow rates in the ml/min range without clogging.

This patterned-coupler approach overcomes key limitations of conventional microfluidic systems by replacing the inherent constraints imposed by narrow microchannels. The elimination of physical microchannels minimizes shear stress on target particles and fundamentally prevents channel clogging. Furthermore, this platform enables a promising solution for high-throughput particle manipulation in the ml/min regime, exceeding the typical flow rates of channel-confined microfluidic systems, which are generally limited to the order of tens to hundreds of μl/min (*57*, *66*).

The proposed platform offers comparable technical capabilities to the existing acoustic tweezer systems while providing distinct advantages in reconfigurability and system simplicity. It enables highly selective spatial control, achieving 2D particle manipulation along intricate, predefined paths using a single source. In contrast to propagating-wave acoustic holography, which relies on complex active array control, the present system achieves reconfigurable channeling through frequency-selective activation of passive resonant couplers (*54*). While evanescent-field-based methods, including metasurfaces and confined Bessel beams, offer strong spatial confinement, their reconfigurability is often limited by fixed structural designs or restricted tuning mechanisms (*55*, *56*). Streaming-assisted manipulation platforms have also demonstrated effective nanoparticle guiding and focusing; however, the field configuration is intrinsically coupled to device geometry and flow conditions, constraining independent control over manipulation pathways (*57*). In this context, the proposed architecture enables scalable and dynamically addressable virtual channel formation in wide-channel environments, while maintaining low system complexity, minimal thermal load, and broad biocompatibility.

While particle trajectories are confined to predefined coupler-paths, the modular nature of the transducer-coupler architecture provides a natural pathway toward enhanced reconfigurability. As discussed above, strategies ranging from frequency-selective excitation to spatially addressable transducer integration offer viable routes to real-time virtual wall reconfiguration. Beyond these approaches, active materials incorporated into the coupler layer itself, such as thermally or electrically actuated soft structures, could dynamically modify the effective coupler geometry, further expanding the accessible field configurations without increasing control complexity (*67*). Last, in contrast to the pronounced heating commonly observed in high-frequency acoustic tweezer applications, the use of low-frequency (200 kHz) ultrasound minimizes acoustic heating, thereby ensuring a highly biocompatible environment.

Furthermore, the design is robust and scalable. By using an acoustic wavelength that is significantly larger than the particle size, we ensure a robust acoustic field with minimal scattering-induced distortion, enabling reliable operation in dense suspensions and under large-volume batch-processing conditions. It is worth noting that our device architecture closely aligns with the BAW tweezers (*68*). This alignment potentially enables large and cost-effective piezoelectric elements to be used for high-precision manipulation, with a much lower frequency compared with conventional surface acoustic wave devices, which are narrow-banded in nature.

The capabilities demonstrated by the collector and separator designs highlight the broad applicability of acoustic virtual walls across biomedical and biochemical particle-handling processes. For instance, the collector geometry is well suited for continuous particle accumulation, offering potential advantages for sample enrichment in diagnostic workflows involving biological particles. While continuous-flow particle accumulation has previously been realized through acoustic streaming-based mechanisms (*57*), the present work achieves confinement via radiation-force-driven localization within a wide-open channel, enabling particle accumulation at significantly higher flow rates. Likewise, the separator design is readily applicable for discriminating particles or cells based on differences in mechanical or acoustic properties, enabling applications such as live/dead cell separation, isolation of circulating tumor cells from peripheral blood, separation of stem cells from differentiated progeny, and sorting of synthetic particles or microcarriers with distinct material compositions (*39*). Depending on the particle types, the platform can be extended to separate multiple particle classes simultaneously by exploiting specifically engineered acoustic-property contrasts.

Future work on this acoustic tweezer concept will focus on designing finer and stronger acoustic virtual walls by maximizing the acoustic radiation force through sharper pressure gradients. One promising strategy is implementing out-of-phase excitation in a duo-coupler design. This specific excitation causes the absolute pressure field to approach zero precisely between the couplers, resulting in a significantly steeper gradient. This characteristic behavior is unique to near-field acoustics; in far-field scenarios, two out-of-phase sources primarily cancel the intensity rather than enhancing the gradient. A steeper gradient not only strengthens the acoustic radiation force but also improves lateral confinement, enabling deep-subwavelength particle manipulation. A second, complementary approach involves advancing the physical waveguide structure using high-resolution additive manufacturing techniques. Precise control over the waveguide thickness and geometry would allow us to tailor evanescent decay profiles and achieve sharper pressure confinement, thereby further enhancing the performance and resolution of the acoustic virtual walls.

The use of a single bulk transducer further highlights the practicality and scalability of the technique across diverse settings, from high-throughput cell sorting and continuous particle enrichment to organoid assembly and multicellular patterning. With continued optimization and integration of reconfigurable elements, the acoustic virtual wall platform offers a scalable and cost-effective foundation for next-generation lab-on-a-chip systems, supporting a broad spectrum of applications including single-cell analysis, biomarker detection, point-of-care diagnostics, drug screening, and programmable biofabrication.

## MATERIALS AND METHODS

### Acoustic radiation force

The mechanism behind the acoustic tweezers is modeled by the acoustic radiation force ($F_{\text{rad}}$) exerted on a particle within a fluid medium. This radiation force is characterized by the scattering of the acoustic field by the particle and is derived from the Gor’kov potential, $U_{\text{rad}}$, such that

$$F_{\text{rad}}=-\nabla U_{\text{rad}}$$ (3)

For a small spherical particle of radius *R* in a lossless fluid, the potential is defined as

$$U_{\text{rad}}=\frac{4\pi }{3}R^{3}\left[f_{1}\frac{\kappa_{l}}{2}\left\langle p_{\text{in}}^{2}\right\rangle -f_{2}\frac{3}{4}\rho_{l}\left\langle v_{\text{in}}^{2}\right\rangle \right]$$ (4)

$$f_{1}=1-\frac{\kappa_{p}}{\kappa_{l}},f_{2}=\frac{2\left(\rho_{p}-\rho_{l}\right)}{2\rho_{p}+\rho_{l}}$$ (5)

where $f_{1}$ is the monopole coefficient (compressibility contrast) and $f_{2}$ is the dipole coefficient (density contrast) (*62*, *63*). Subscripts p and l denote the particle and liquid properties, respectively. In many microfluidic applications, the similarity in density between the target particles and water means the acoustic pressure field governed by $f_{1}$ is the major factor in manipulating the particles inside the chamber.

### Device fabrication and set-up

The PDMS layer is fabricated with the standard PDMS molding process. The negative mold was designed in SolidWorks and printed on a stereolithography 3D printer (ELEGOO, Saturn 3 Ultra) (*32*, *43*). The mold was washed with 99% isopropyl alcohol, ultraviolet-cured for 1 hour, and baked at 100°C for 4 hours to remove residual curing inhibitors from the negative mold (*69*). Part A and B of the PDMS (Dow Corning, Sylgard 184) are mixed thoroughly at a 10:1 weight ratio with added white dye, degassed in a vacuum chamber, and then poured into the mold. The mold is degassed again, then baked in an oven at 60°C for 4 hours to cure the silicone. The PDMS is then separated from the mold and attached to the prefabricated drilled glass plate with a plasma treatment (Harrick Plasma, PDG-32G). The bottom PDMS spacer was molded as mentioned above and plasma-treated on the bottom surface of the PDMS pattern.

The experimental platform was assembled by mounting the fabricated composite layers onto a customized power transducer with a center frequency of 200 kHz and a built-in electrical impedance matching circuit and acoustic impedance matching layer (GZ Doppler Electronic Tech Inc.). The transducer was driven by a continuous 200-kHz sinusoidal signal (400 to 500 mVpp) generated using a function generator (Rigol Technologies, DG4102) and subsequently amplified by +50 dB using an RF power amplifier (E&I 2100L). The corresponding electrical output power of the amplifier ranged from 9 to 20 W. For fluidic transport, microparticles (Cospheric LLC, 250-μm fluorescent polystyrene particles) were introduced into the wide-open channel using a peristaltic pump (CHONRY, BT100MH). The resulting particle trajectories and manipulation dynamics were characterized and recorded using a stereo microscope equipped with a digital camera (AmScope, SM-3T). The surface temperature of operating system was measured with a thermal camera (TOPDON, TC-002C).

### PDMS microparticle fabrication

We fabricated microparticles of PDMS (Dow Corning, Sylgard 184) inspired by the procedure in “Holograms for Acoustics” (*53*). The PDMS base (premixed with red ink) and curing agent are mixed thoroughly at a 10:1 weight ratio and added to a water bath containing 1 weight % nonionic surfactant (Sigma-Aldrich, Pluronic F-127). The emulsion was heated at 90°C and continuously agitated using a magnetic stirrer for 1 hour. The cooled mixture then passes through a 250-mesh sieve to keep particles smaller than 300 μm.

### Numerical simulations

The mode shape is analyzed using eigenfrequency simulation in COMSOL Multiphysics 6.3 (COMSOL Inc.), using the Pressure Acoustics, Solid Mechanics, and Particle Tracing modules. The Pressure Acoustics module simulates the pressure field inside the water and PDMS layers, with a Plane Wave Radiation condition applied on the left and right sides of the simulation domain, while soft boundaries were applied around the air domains. The Solid Mechanics module was applied to the top glass layer to study the reflected fields from the solid layer. We then investigated the actual pressure distribution in the frequency domain around the resonance. The Particle Tracing module visualized the routing of PDMS particles for frequency-based tunable reconfiguration of acoustic virtual wall, applying particle diameters of 250 μm and acoustophoretic forces coupled with precalculated acoustic field.
