# Supplementary material for: Acoustic virtual walls enable open, scalable, and programmable microfluidics
Source: Sci Adv. 2026 Aug 14;12(33):eaec0104. doi: 10.1126/sciadv.aec0104 (PMC13475609; doi:10.1126/sciadv.aec0104)
Supplement: Supplementary file 1 [file sciadv.aec0104_sm.pdf]

Science Advances
AAAS

Supplementary Materials for

Acoustic virtual walls enable open, scalable, and programmable microfluidics

Hajin Oh *et al.*

Corresponding author: Junfei Li, junfeili@purdue.edu

Sci. Adv. **12**, eaec0104 (2026)
DOI: 10.1126/sciadv.aec0104

The PDF file includes:

Supplementary Text S1 to S3
Figs. S1 to S7
Table S1
Legends for movies S1 to S4

Other Supplementary Material for this manuscript includes the following:

Movies S1 to S4

Text S1 The calculation of acoustic pressure field

The harmonic plane wave in a 2D waveguide system was calculated by specifying the acoustic pressure field in each domain and applying the boundary conditions. Using the general wave equation, the pressure field can be expressed as

$$p = \sum_{k_x} \sum_{k_z} A_{xz}\, e^{-jk_x x} e^{-jk_z z} \tag{S1}$$

where the total wavenumber satisfies $k^2 = k_x^2 + k_y^2 + k_z^2$. In the ideal design, k_y is assumed to be zero, as there is no wave propagation in the y-direction.

The waveguide system is partitioned into seven domains as illustrated in Fig. S1, and the pressure field in each section can be calculated individually. The origin of the coordinate system is assumed to be at the center of the coupler aperture.

The pressure field inside the first section can be described as follows in the lowest order mode ($k_x = \pi/w$) with the definition of wavenumber ($k = \omega/c$):

$$p_1 = cos\left(\frac{\pi x}{w}\right)\left(A_{z1} e^{-jk_{z1}z} + B_{z1} e^{jk_{z1}z}\right) \tag{S2}$$

$$k_{z1} = \sqrt{\frac{\omega^2}{c_{pdms}^2} - \frac{\pi^2}{w^2}} \tag{S3}$$

where c_{pdms} is the speed of sound in PDMS. Here, the vertical wave number k_{z1} is governed by the waveguide width w. As the waveguide width decreases, k_{z1} becomes imaginary, indicating exponential decay of the acoustic field and suppression of vertical wave transmission.

In sections 2 and 3, we are interested in the laterally confined mode, which requires the wave to be evanescent in the $-x$ direction. By applying air boundary conditions at the bottom of Domain 2 ($z = 0$) and the solid boundary at the top of Domain 3 ($z = d_2$), the corresponding pressure fields in both domains can be calculated. The field components are:

$$p_2 = A_2 e^{\alpha x} \sin(k_{z2} z) \tag{S4}$$

$$p_3 = A_3 e^{\alpha x} \cos\big(k_{z3}(z - d_2)\big) \tag{S5}$$

where α represents the lateral evanescent decay coefficient. Pressure and particle velocity continuity boundary conditions are further applied to determine the unknown coefficients (A_{z1}, B_{z1}, etc.). Assuming α is real and positive, the lateral wavenumber can be expressed as $k_x = i\alpha$, where $\alpha^2 = k_{z,i}^2 - k_i^2$ ($i = 2{,}3$). From these relations, the wave field in the x-direction is controlled by the thickness of each material (d_1 and d_2). As d_1 and d_2 decrease, $k_{z,i}$ increases, leading to a larger exponential decay of the pressure field in the $-x$ direction (evanescent field). The spatial confinement of the field is therefore governed by the exponential decay rate, which can be controlled through the geometric parameters, d_1 and d_2.

The harmonic wave equations for the remaining regions can be constructed accordingly. Domains 6 and 7 are treated as symmetric counterparts of Domains 2 and 3. Equation sets for Domains 4 and 5 will include multiple unknowns which can be found by applying the continuity of pressure and velocity at the interfaces. Alongside the pressure equations, the particle velocity is calculated as $u = -\frac{1}{j\omega\rho}\nabla p$. Since the resultant set of equations is transcendental, a numerical method is utilized to determine the pressure field throughout the system.

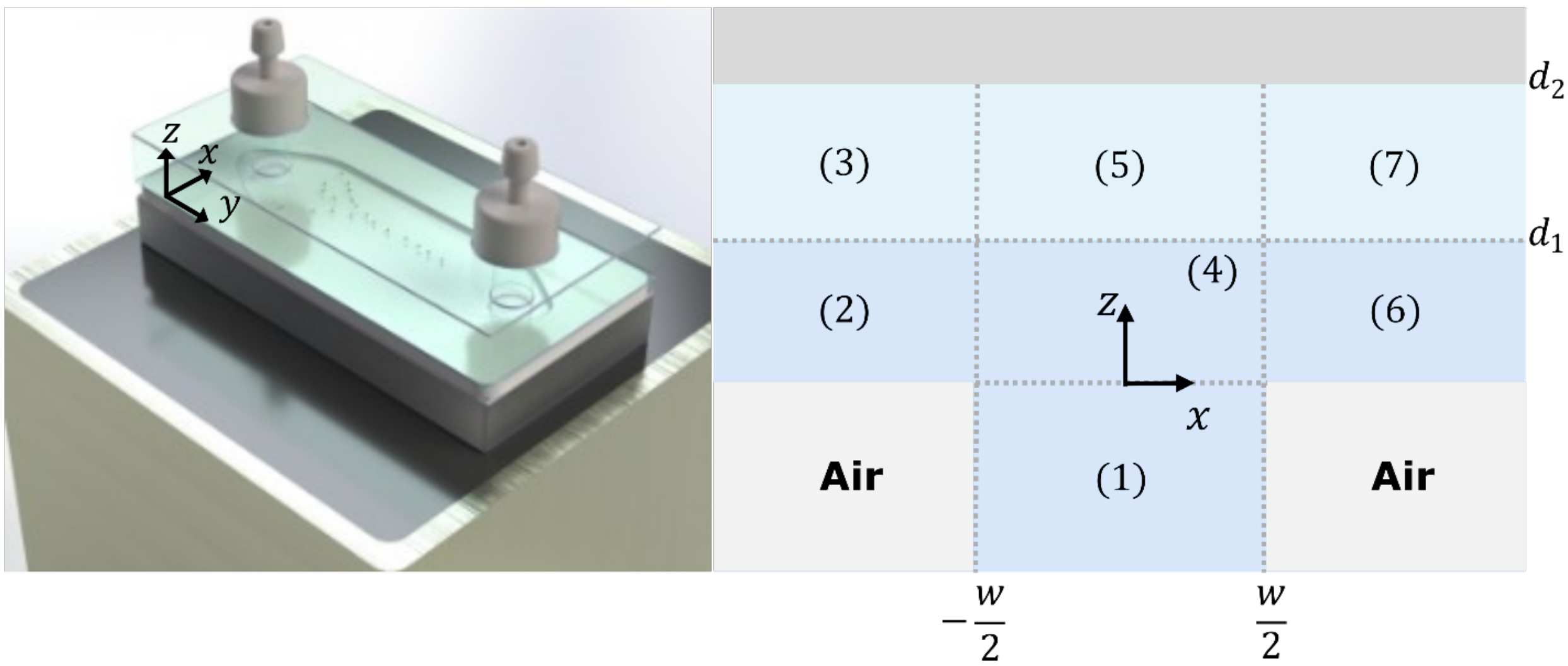

Figure S1 | Cross-sectional view of the waveguide and coupler layers in *xz*-plane, partitioned into seven computational domains. The domain segmentation is used to define material properties, boundary conditions, and governing equations for the acoustic field analysis.

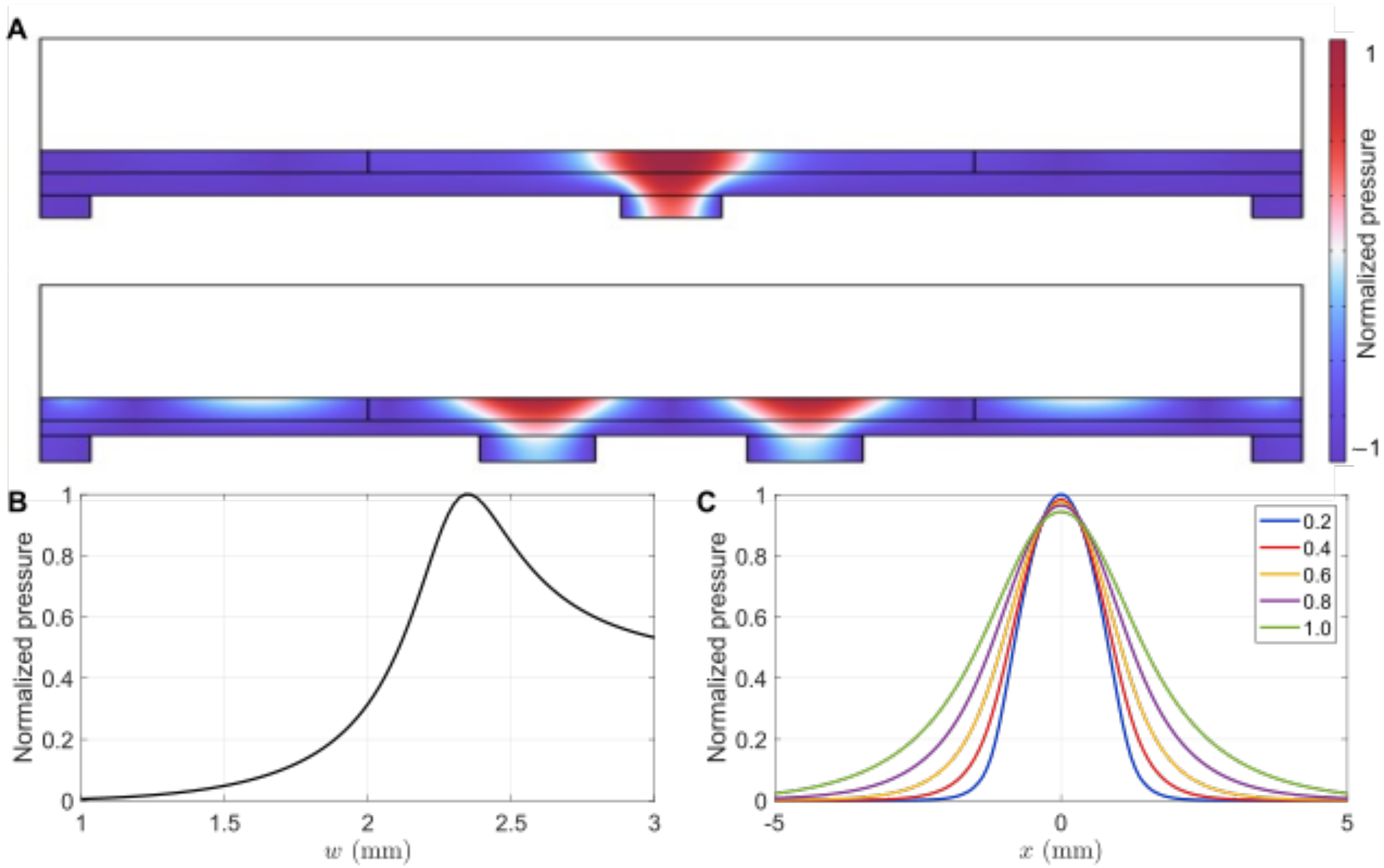

Figure S2 | Numerical simulations of both single- and double-coupler systems. (A) Modal analysis of single- and double-coupler configurations. (B) Normalized absolute pressure in the water chamber as a function of coupler width, w. (C) Normalized acoustic pressure as a function of the PDMS membrane thickness, t_2, demonstrating a sharper pressure gradient with thinner membrane thickness.

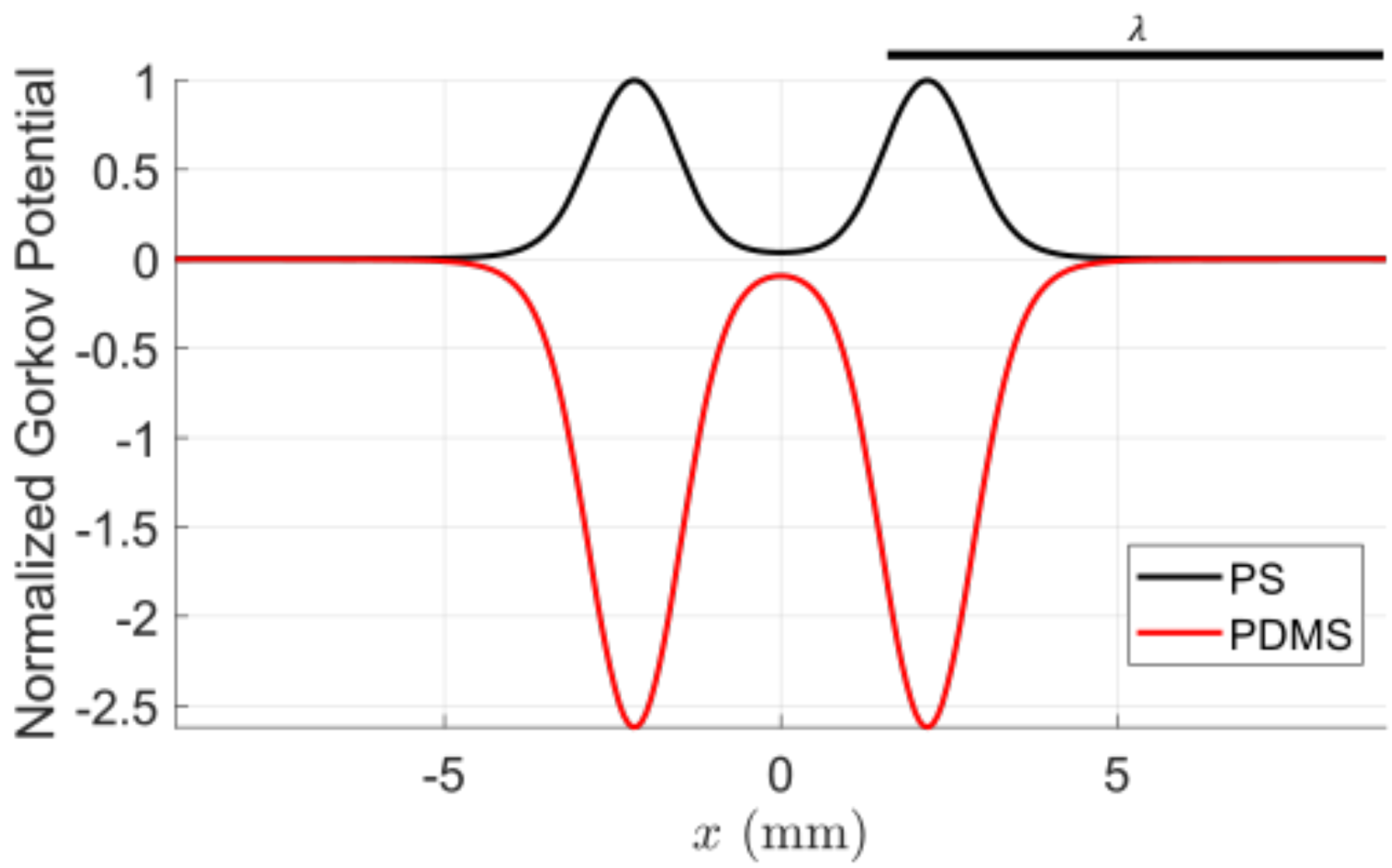

Figure S3 | Gor'kov potential of two particles, PS and PDMS, with the representative diameters used in the experiments interacting with double-coupler design, illustrating material-dependent trapping behavior within the acoustic virtual wall.

Text S2 Resonant design for spacer

The spacer was incorporated to mitigate the near-field effect on the transducer surface. In the near-field region, the acoustic pressure distribution is inherently nonuniform, which disrupts consistent wave propagation into the microchannel. By introducing a PDMS spacer of 6 mm thickness, the wavefront becomes more spatially uniform before reaching the patterned membrane layer.

The spacer height was determined through parametric studies considering the acoustic properties of PDMS (acoustic velocity: 1002 m/s) at the operating frequency of 200 kHz, corresponding to a wavelength of ~5.01 mm in PDMS. The 6 mm spacer thickness was selected to ensure sufficient distance from the near-field region while maintaining constructive pressure buildup within the system.

The patterned PDMS membrane was bonded to the spacer via plasma treatment, forming enclosed air pockets. Due to the high impedance mismatch at the PDMS-air interface, acoustic energy is selectively transmitted only through the open aperture regions, resulting in locally concentrated evanescent pressure fields at the channel interface, as confirmed by the finite element simulation (Fig. S4).

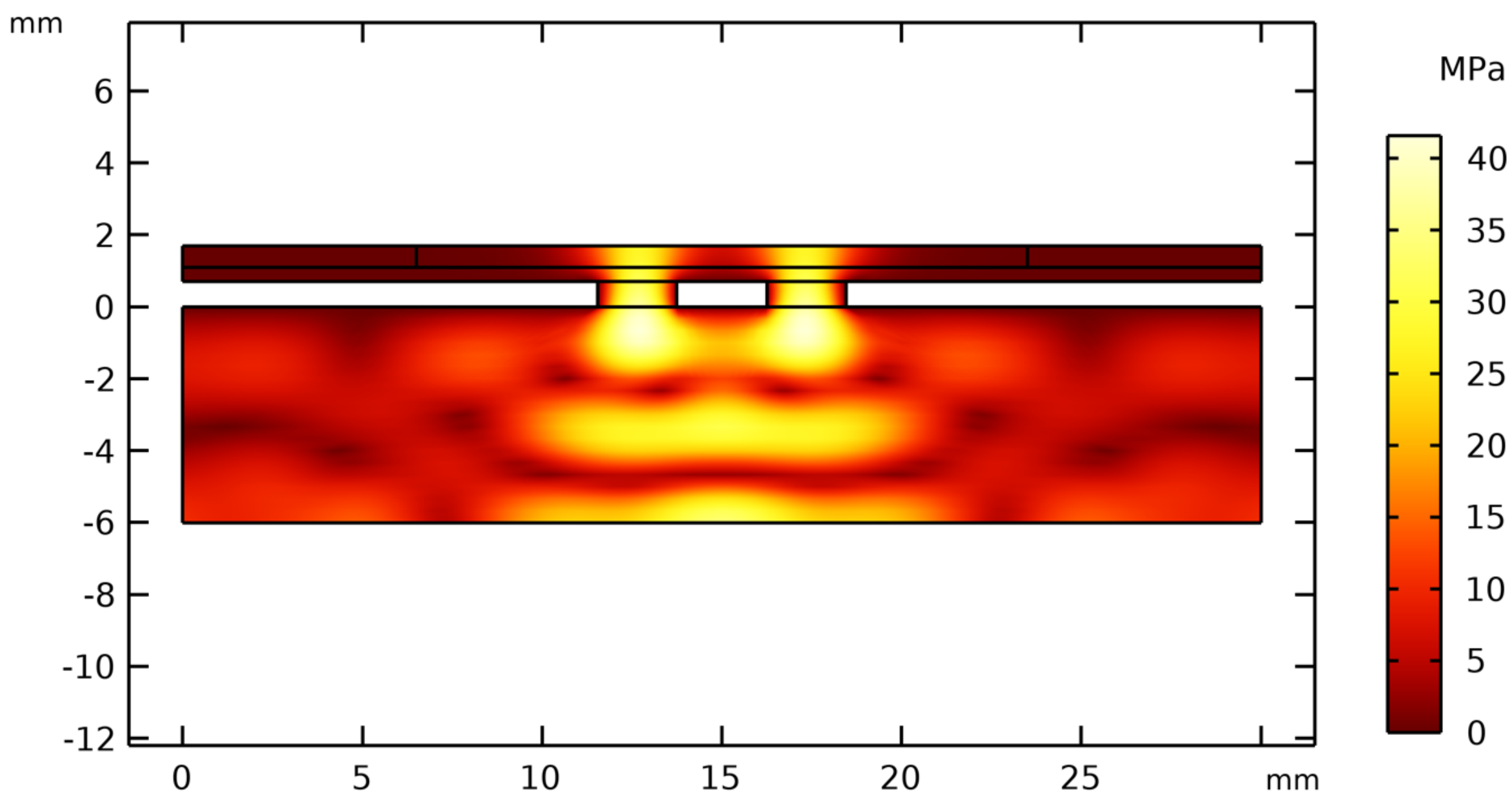

Figure S4 | Finite element simulation of the acoustic pressure field in the spacer-integrated system. Acoustic pressure distribution of the system consisting of a 6 mm PDMS spacer, patterned coupler layer with enclosed air pockets, and the water channel.

Text S3 Calculation of critical radius

The critical radius quantifies the force balance between the acoustic radiation force, which scales with the cube of the particle radius (R^3), and streaming-induced drag force, which scales linearly with particle radius (R). The calculated force ratio between acoustic radiation and streaming forces in the present system was 4.4×10^{-4}, which was used to calculate the critical radius. This ratio remained approximately constant over 200 ± 5 kHz, which resulted in a critical radius of $R_{critical} \approx 2.62\ \mu m$, where R = 125 μm corresponds to the particle radius used in the force evaluation

$$F_{rad} = F(f_1, f_2, \dots)R^3 \tag{S6}$$

$$F_{streaming} = F(\mu, U_{as})R \tag{S7}$$

$$R_{critical} \approx \sqrt{\frac{F_{streaming}}{F_{rad}}}R \tag{S8}$$

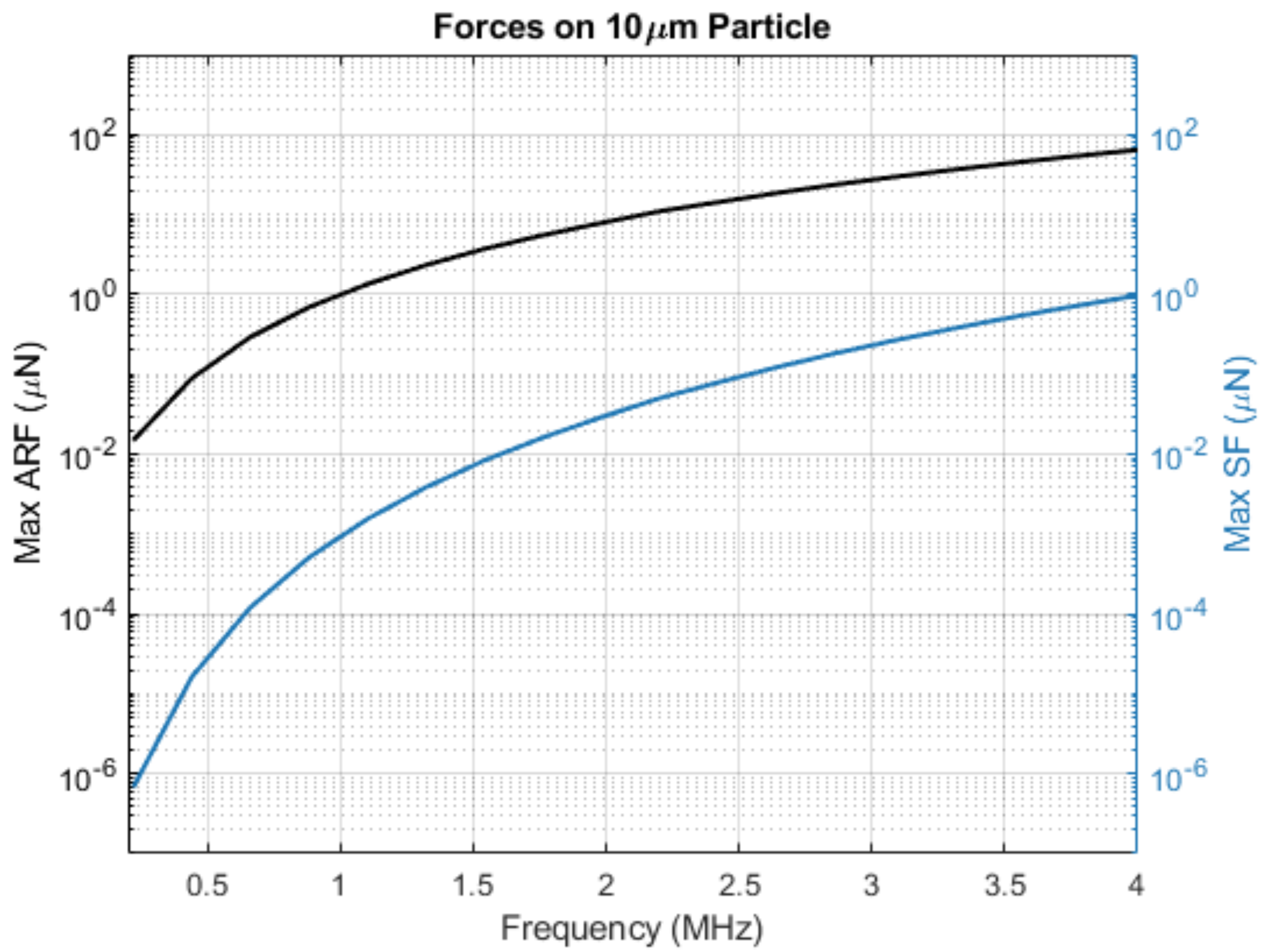

Figure S5 | Acoustic radiation and streaming forces calculated in a higher-frequency configuration. In the simulations, the coupler dimensions (width and height) and water channel height were proportionally scaled down (from $\times 1$ to $\times 1/20$) to maintain a consistent evanescent acoustic field, while channel width was fixed at 12 mm. The forces were evaluated on a 10 μm particle, reflecting the reduced channel thickness at higher frequencies. The results show that the acoustic radiation force increases with frequency and remains dominant over the streaming-induced force. It is worth noting that the channel width is maintained, resulting in a coupler-to-channel width ratio of approximately 1:120.

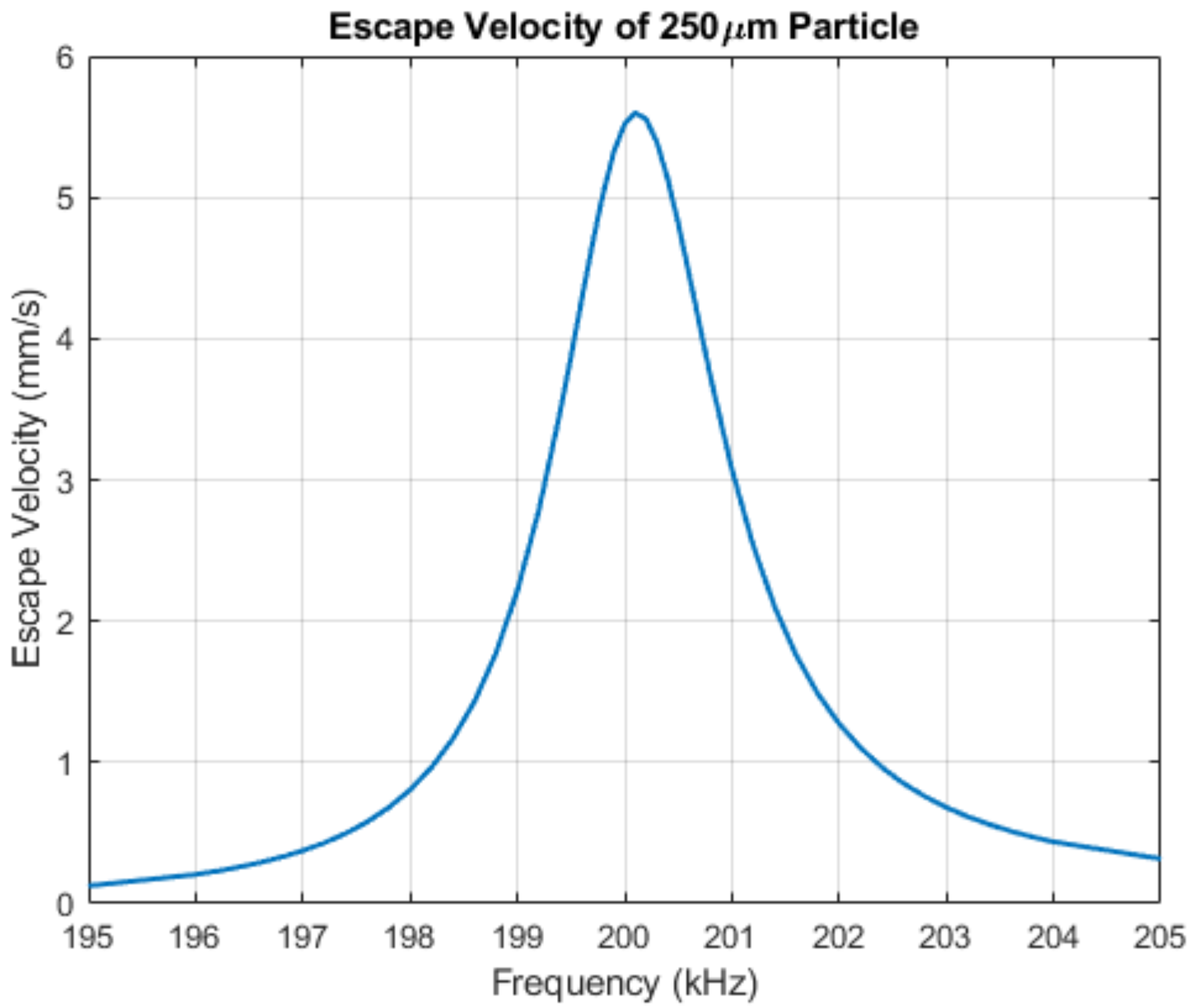

Figure S6 | Escape velocity of 250 μm particle as a function of frequency. Based on the numerically calculated acoustic radiation force around the operating frequency, the escape velocity was determined by the force balance between the acoustic radiation force and hydrodynamic drag force. The resulting velocity was scaled using the experimentally measured escape velocity of 5.6 mm/s.

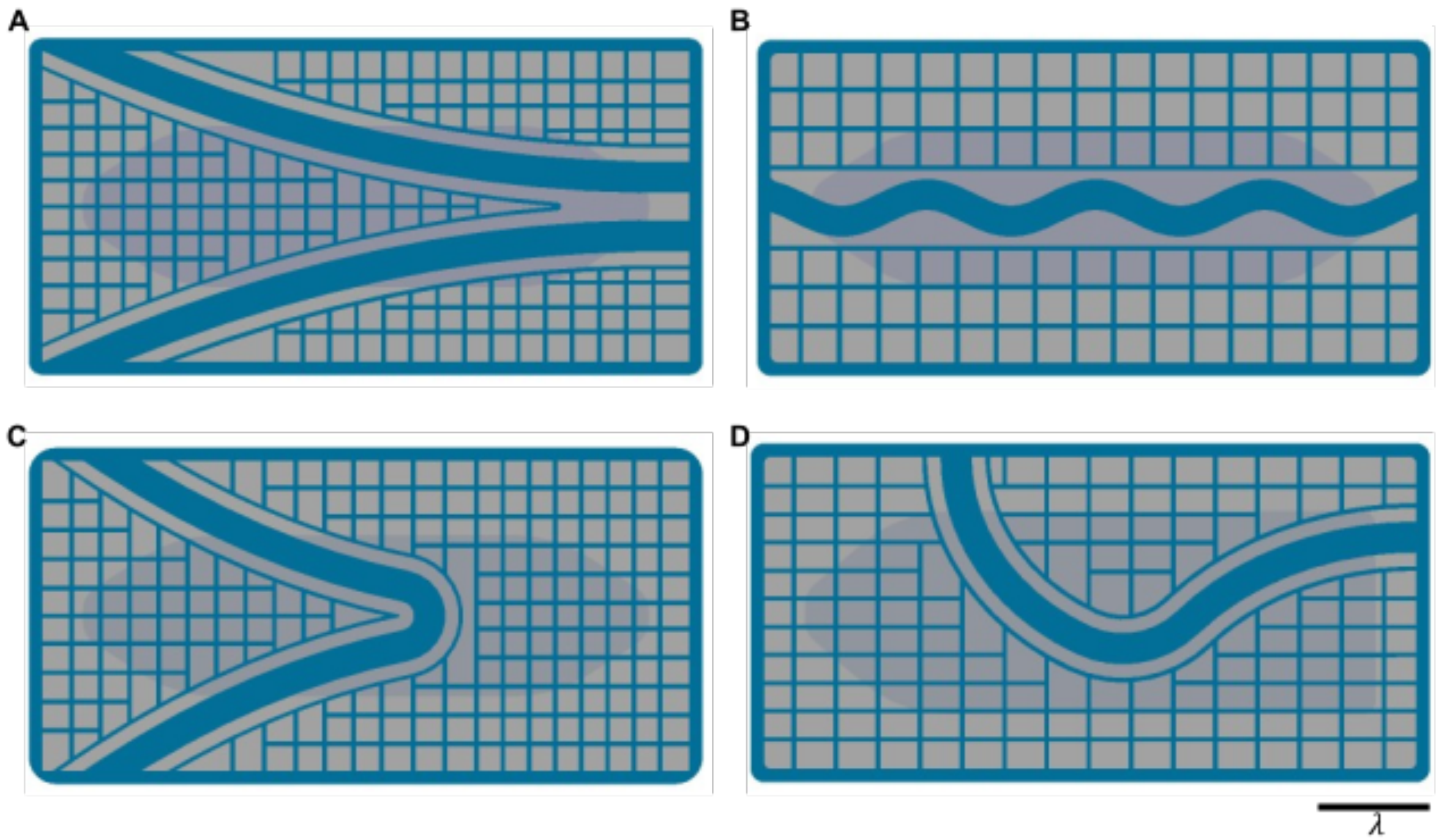

Figure S7 | Three-dimensional geometry of coupler patterns with added mechanical supports. (A) Funnel-like channeling for PS particles. (B) Meandering channeling for PDMS particles. (C) Tunable collector for PS particles. (D) Binary separator.

Table S1. Comparison of acoustic particle manipulation platforms

Platform	Frequency	Spatial Resolution	Reconfigurability	Wide channel	Thermal load	System Complexity	Flow rate regime
**This work (Acoustic Virtual Wall)**	**200 kHz**	**$\sim\lambda/4$ subwavelength**	**Frequency-selective**	**Yes**	**Low (Low frequency)**	**Single transducer**	**mL/min**
Propagating-wave acoustic holography (*54)*	~500 kHz-MHz	Diffraction-limited	High (50,000 fps)	Yes	Moderate	64-element arrays	Not demonstrated
Evanescent Bessel beam (*56*)	Sub–MHz	Subwavelength	Structurally defined	No – confined	Moderate	Single transducer	Not demonstrated
Reconfigurable acoustic metasurface (*55*)	< 2 MHz	Subwavelength	High (Real-time)	No – confined	Low-moderate	Membrane array	μL/min
Membrane waveguide (MAWA) (*58*)	Low-mid MHz	Subwavelength	Mode-selective	No – confined	Low	Low	nL- μL/min (streaming-driven)
GHz BAW virtual microchannel (*57*)	~ 2 GHz	Nanoscale (acoustic streaming)	Typically Limited	No – Typically confined	Potentially elevated	Thin-film BAW	nL- μL/min
Resonant BAW (*50*, *51*)	1 – 10 MHz	$\lambda/2$ (channel width)	Low – fixed by mode	No	Low – moderate	Single transducer	μL-mL/min

Supplementary Movie S1. Funnel-like channeling of PS particles within the wide-open chamber.

Supplementary Movie S2. Meandering channeling of PDMS particles following a curved trajectory within the wide-open chamber.

Supplementary Movie S3. Tunable collection and release of PS particles within the wide-open chamber.

Supplementary Movie S4. Sorting of PS and PDMS particles using a single curved trajectory within the wide-open chamber.

Note: Due to the intrinsic pulsation of the peristaltic pump, particles intermittently decelerate or momentarily stop during transport.
